# MEN2: surgical precision in the era of precision medicine

**DOI:** 10.1530/ERC-24-0251

**Published:** 2025-06-06

**Authors:** Tom R Kurzawinski, Colin R Butler, Tarek Abdel Aziz

**Affiliations:** ^1^ Consultant Endocrine Surgeon, University College Hospital and Great Ormond Street Hospital for Children NHS Trusts and Honorary Assistant Professor University College London, London, UK; ^2^ Consultant ENT Surgeon, Department of Paediatric Otorhinolaryngology, Great Ormond Street Hospital, Institute of Child Health, University College of London, London, UK; ^3^ Consultant Endocrine Surgeon, University College Hospital NHS Trust and Honorary Assistant Professor University College London, London, UK

**Keywords:** medullary thyroid cancer, phaeochromocytoma, primary hyperparathyroidism, MEN2, surgery

## Abstract

**Graphical abstract:**

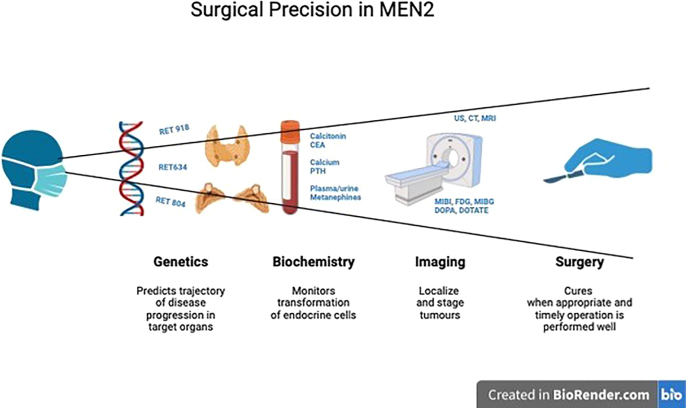

**Abstract:**

Medullary thyroid cancer, phaeochromocytoma and primary hyperparathyroidism in patients with multiple endocrine neoplasia type 2 can all be cured by surgery on the condition that they are detected early before locoregional or distant spread of malignant disease occurs and long term metabolic and structural damage to cardiovascular, renal and skeletal systems takes place. Recent scientific discoveries and technological advances made surgical decision process more precise and facilitated personalised treatments. *RET* analysis enables us to see this syndrome not as a monolith but as a cluster of different phenotypic presentations, each sending patient on an individual journey, which can be anticipated but not determined. Biochemical monitoring provides regular updates on transformation of endocrine cells in target endocrine organs and together with imaging helps to decide on time and extent of surgery. Advances in surgical technology allow for safer and less invasive interventions resulting in fewer complications, less trauma and better functional outcomes. Calibrating magnitude of surgery able to cure but do minimal harm, timing and performing it well is the art of the surgical precision in MEN2 patients. Surgical outcomes have improved in the last 30 years and we need to continue on this road. Precision in surgery aiming at near perfect surgical performance is achievable and this review looks at surgical decision making process through the prism of genetics and biochemical testing combined with imaging, former setting a trajectory for the disease progression with a fair degree of probability and latter assessing functional and structural changes over time.

## Introduction

Medullary thyroid cancer (MTC), phaeochromocytoma (PHAEO) and primary hyperparathyroidism (PHPT) can all be cured by surgery on the condition that they are detected early before locoregional or distant spread of malignant disease occurs and long term metabolic and structural damage to cardiovascular, renal and skeletal systems takes place. Clinical description and pathological characterisation of these tumours (1886–1959) as isolated entities arising in a sporadic fashion was soon followed by development of effective surgical treatments (Frankel 1886, Pick 1912, Albright *et al.* 1934, Hazard *et al.* 1959). It was not until early 1960’s, when astute clinical observations led to the discovery that these tumours can appear as a cluster in certain individuals and that predisposition to develop them can be inherited in autosomal dominant fashion with a 50% risk of passing it on to the offspring (Sipple 1961, Cushma 1962). The diagnosis of hereditary syndrome of the multiple endocrine neoplasia type 2 (MEN2) characterized by its phenotypic features was established (Steiner *et al.* 1968).

However, correct diagnosis and timing of surgery remained a challenge as it was uncertain which children of a parent with MEN2 inherited this condition, tumours developed at different ages and sometimes not at all. Scrupulous follow up and waiting for tumours to manifest themselves was the only therapeutic strategy available. Clinical management was soon refined by the application of relevant biochemical markers such as calcitonin, catecholamines and parathormone facilitating earlier diagnosis and defining cure or disease progression (Wells *et al.* 1978, Gagel *et al.* 1988, Easton *et al.* 1989). This was a first step towards precision management of MEN2 but the real revolution arrived in the early 1990’s with the pivotal discovery of *RET* as the causative gene (Takahashi *et al.* 1985, Donis-Keller *et al.* 1993, Mulligan *et al.* 1993). Linking RET to MEN2 was followed by the rapid accumulation of knowledge about genotype–phenotype correlation, which proved to be one of the strongest known in clinical practice (Mulligan *et al.* 1994, Machens *et al.* 2003, 2024). The era of modern precision medicine, based on accurate genetic diagnosis and prediction of development of some tumours at certain age began. Although genetics was able to precisely identify RET carriers, environmental, second germline or somatic mutations and other genetic or epigenetic influences made precise timing of pathological events in thyroid, adrenal and parathyroids difficult. Genetics alone could not therefore be the only factor influencing decision about timing and extent of surgery (Eng & Plitt 2023, Wells *et al.* 2015, Machens & Dralle 2024).

This review looks at surgical decision making process through the prism of genetics and biochemical testing combined with imaging, former setting a trajectory for the disease progression with a fair degree of probability and latter assessing functional and structural changes in target endocrine organs over time. It also reviews recent advances in surgery facilitating innovative approaches to operations on thyroid, adrenals and parathyroids and their impact on outcomes. We performed a narrative literature review of all articles published in English between 1993 and 2024 with a focus to select papers which had the greatest impact on the evolution of surgical practice in managing patients with MEN2 and also included older papers of historical relevance going back to 1934.

## Medullary thyroid cancer in MEN2

MTC is the pathognomonic tumour in MEN2 and almost all patients with this syndrome will develop it in their lifetime (Eng & Plitt 2023, Wells *et al.* 2015). It is derived from the parafollicular C-cells located predominantly laterally in upper 2/3 of the thyroid (Livolsi 1997). Its age dependent penetrance, predetermined by the type of *RET* pathological variant, starts with normal C cells developing hyperplasia (CCH), a pre-malignant condition which progresses to MTC. This process of malignant transformation is driven by oncogenic activity of germline *RET* mutations. Invasion of lymphatic and blood vessels leading to metastases to locoregional lymph nodes and distant organs, mostly liver, bones, brain and lungs, defines its aggressiveness and possibly requires additional somatic mutations (Leboulleux *et al.* 2002, Voss *et al.* 2017, Raue *et al.* 2018, Castinetti *et al.* 2019, Raue *et al.* 2019, Machens *et al.* 2023).

Historically, diagnosis of MTC was the commonest first presentation in a family lineage but it was the PHAEO which presented the greatest risk of death to patients with MEN2 (Machens *et al.* 2023). Nowadays, although earlier diagnosis facilitated by genetic testing and biochemical screening often leads to thyroidectomy before malignant transformation and metastases, metastatic MTC remains the main cause of morbidity and mortality in MEN2 and its management presents the greatest challenge in treating patients with this syndrome (Castinetti *et al.* 2019, Shankar *et al.* 2021, Machens *et al.* 2023).

## Genetics

Twenty-five percent of all MTC cases are hereditary and MEN2, encompassing MEN2a, 2b and FMTC, is the only syndrome associated with familial form of this cancer (Wells *et al.* 2015, Gild *et al.* 2023). Phenotype-genotype correlation and age dependent penetrance of MTC, in comparison with other target organs and development of PHAEO and PHPT, is the strongest and best understood pathological process in MEN2 (Machens *et al.* 2024).

ATA Guidelines stratify *RET* pathogenic variants into highest, high and moderate risk groups, although some advocate splitting moderate risk into two further subgroups of higher and lower risk mutations (Brandi *et al.* 2001, Wells *et al.* 2015, Machens *et al.* 2018*b*, Machens & Dralle 2024). Definition of risk in the context of MTC is concerned with development and timing of CCH and its malignant transformation but it remains contentious whether it also reflects MTC aggressiveness, which might require additional genetic aberrations (Leboulleux *et al.* 2002, Yip *et al.* 2003, Voss *et al.* 2017, Raue *et al.* 2019, Machens *et al.* 2021*a*). Other *RET* variants of unproven pathogenicity are classified as variants of unknown significance and will not be discussed in this review.

*Highest risk group*, associated with *RET* 918 pathogenic variant is responsible for 95% of MEN2b cases, the most severe phenotype of MEN2 syndrome. It is a rare disease (prevalence 0.9–1.65 per million) with the earliest onset of CCH and documented cases of malignant transformation to MTC within first year of life and early lymph node metastases (Zenaty *et al.* 2009, Castinetti *et al.* 2019). Majority of cases (75–90%) are *de* *novo* mutations and early diagnosis, in the absence of family history, is difficult (Brauckhoff *et al.* 2008, Machens *et al.* 2013, Mathiesen *et al.* 2017, Elisei *et al.* 2019). Patients with MEN2b have strong extra-endocrine manifestations and identifying them correctly is the best chance to raise the possibility of MEN2b diagnosis early, which then can be verified by genetic test (Brauckhoff *et al.* 2008, Castinetti *et al.* 2019). Early diagnosis however is challenging as signs which can develop within first year of life are subtle (alacrimia) or non-specific (feeding problems, constipation or megacolon) and more recognisable features such as mucosal neuromas and a marfanoid body habitus may not become clinically apparent until several years of age (O'Riordain *et al.* 1993, Eng *et al.* 1996, Cohen *et al.* 2002, Brauckhoff *et al.* 2008, Vasen *et al.* 1992).

*High-risk group* is associated with C634 and A883 *RET* pathological variants, former being the commonest cause responsible for 90–95% of MEN2a and latter a rare and milder form of MEN2b (Wells *et al.* 2015). Patients with C634 pathogenic variant develop CCH within first years of life and malignant transformation occurs between 5 and 10 years, although MTC has been reported in a 3 year old child (Wells *et al.* 2015, Al-Kurd *et al.* 2018). Time lag between development of MTC and progression to lymph node metastases is around 14 years (13.6–14.5). (Machens *et al.* 2021*b*).

*Moderate risk group* patients have *RET* mutations in exons 8, 10, 11, 13, 14, 15 and 16 and pathogenic variants in cysteine codons 609, 611, 618 and 620 in exon 10 are responsible for 10% of MEN2 cases and 50% of FMTC cases (Takahashi *et al.* 1985, Hansford & Mulligan 2000). They have low transforming activity and CCH and MTC occur usually later in the third decade and tumour progresses to lymph node metastasis around 9 years of life (8.6–9.1) (Machens *et al.* 2021*b*). However, disease expression in moderate risk group can be very variable, even within the same family lineage. MTC development within the same family (C609) varied between 9 and 48 years and death from metastatic disease was reported in a 12 year old child with RET 804 mutation (Frohnauer & Decker 2000, Mian *et al.* 2009).

## Biochemistry and imaging

*Biochemical assessment* provides insight into changes of C cells function and mass, and because phenotypic variability makes precise genetic prediction of the CCH onset, malignant transformation to MTC and development of metastases difficult, it supplements genetic information and helps to evaluate progress of the disease over time. C cells are neuroendocrine cells and as such can produce an array of hormones, and calcitonin (Ct and carcinoembryogenic antigen (CEA) are best biomarkers to monitor their transformation (Elisei *et al.* 2012, Wells *et al.* 2015, Gild *et al.* 2023).

Calcitonin can be measured with high sensitivity and specificity by the immunochemiluminometric (ICMA) assays, but normal ranges for individual commercial ICMA platforms differ, and therefore it is important to use the same assay for continuous monitoring. Calcitonin is not detectable in at least half of the normal population, and ranges of calcitonin are different for males and females (<11.7 ng/L, <5.2 ng/L, Immulite 2000 XPi; Siemens Diagnostics, Germany). Calcitonin levels are highest in the first year of life (48.9–75 ng/L), gradually decline in 2nd (15 ng/L), and, starting from the third year of life, they resemble values in adults (Basuyau *et al.* 2004, D'Herbomez *et al.* 2007, Castagna *et al.* 2015).

Gradually increasing levels of calcitonin in *RET* carriers reflects development of CCH, and calcitonin crossing upper ranges of normal is thought to be an early indicator of malignant transformation, although micro MTC was found in patients with normal calcitonin (Skinner *et al.* 1996). Abnormal calcitonin levels are associated with increased risk of locoregional lymph node metastases (53–500 ng/L), and distant metastases are likely with calcitonin >1,000 ng/L (Danila *et al.* 2019, Costante & Meringolo 2020). No lymph node metastases were found in children with calcitonin levels below 30 (Rohmer *et al.* 2011) or 40 ng/L (Machens *et al.* 2005).

Calcitonin is a useful arbiter of surgical cure, persistent, recurrent or progressive disease, and response to chemotherapy (Skinner *et al.* 1996, Prete *et al.* 2018, Shankar *et al.* 2021). Undetectable calcitonin indicates cure as non-secretory MTC is extremely rare, and above normal and rising levels signify recurrence and disease progression (Gambardella *et al.* 2019, Gild *et al.* 2023). Fluctuating but within normal ranges concentration of calcitonin after surgery is indicative of failure to eliminate all C cells, but the risk of developing MTC by these patients is uncertain (Pellegriti *et al.* 2003, Prete *et al.* 2018, Prete *et al.* 2023).

Provocative calcitonin testing with calcium or pentagastrin stimulation was historically an important part of diagnosing MEN2 early, as sensitivity of stimulated calcitonin was higher than basal levels. However, in the post-*RET* era, when negative genetic test gives 97% certainty of correct diagnosis, this is rarely necessary (Gild *et al.* 2023). Provocative testing could be unpleasant (especially for children) and potentially dangerous (side effects), and although it is still occasionally used to help with timing of surgery or diagnosing recurrent disease, it is not certain whether it provides additional value to high sensitivity ICMA assay (Elisei *et al.* 2013, Wells *et al.* 2015).

Clinical reliability of calcitonin could be compromised by its instability at room temperature, significant variations between assays, concentration-dependency and biphasic half-life. Procalcitonin (ProCT) has better preanalytical and analytical characteristics and has been suggested as an alternative biomarker of MTC. Recent meta-analysis of 11 studies comparing Ct and ProCT demonstrated its clinical utility in diagnosing and monitoring sporadic MTC, yet to be proven for the patients with MEN2 (Algeciras-Schimnich *et al.* 2009, Giovanella *et al.* 2021). High ProCT to Ct ratio was also found to be correlated with progressive disease and shorter DFS (Algeciras-Schimnich *et al.* 2009).

CEA, not a specific biomarker of MTC, is not elevated in the initial stages of CCH and malignant transformation, does not respond to provocative testing and cannot therefore be relied on when deciding about timing of surgery (Wells *et al.* 1978). CEA is useful for monitoring disease in patients with persistent or recurrent disease, high and rising levels indicating disease progression. Unexpected low levels in patients with advanced disease could be the sign of dedifferentiation, increased aggressiveness and poor prognosis (Bockhorn *et al.* 2004, Frank-Raue *et al.* 2013). Doubling time (DT) of both calcitonin and CEA are predictive of disease progression and are of prognostic value, with DT < 6 months predicting shorter survival and longer DT (>2 years) associated with better prognosis (Barbet *et al.* 2005, Laure Giraudet *et al.* 2008, Laure Giraudet *et al.* 2008, Meijer *et al.* 2010, Ito *et al.* 2016).

*Imaging assessment*, both structural and functional, has an important role in preoperative and postoperative evaluation of MEN2 patients undergoing surgery for MTC. Neck ultrasound before surgery aims to provide information on presence of nodules, their size and distribution within thyroid parenchyma, as well as assessment of locoregional lymph nodes and potential metastatic spread into them. It is helpful in staging and evaluating volume of MTC before therapeutic thyroidectomy but should not guide the timing of prophylactic thyroidectomy in paediatric patients (Morris *et al.* 2013, Wells *et al.* 2015).

Structural assessment with computed tomography (CT) and magnetic resonance imaging (MRI) scans of the neck, chest and abdomen is valuable in patients with suspected locally advanced or metastatic disease and helps to determine extent of surgery (Wells *et al.* 2015, Klain *et al.* 2022).

Functional imaging, often combined with structural scans, improves detection of persistent, recurrent and metastatic disease. Radiotracers used most in imaging of MTC are somatostatin receptor analogues (especially ^68^Ga DOTATE), ^18^FDG and ^18^F-DOPA and their sensitivities for lesion detection vary between 16 and 88% (Ozkan *et al.* 2015, Lee *et al.* 2020, Sahin *et al.* 2020, Gild *et al.* 2023). It is generally acknowledged that ^68^Ga DOTATE has better sensitivity than ^18^FDG in lesion detection (despite low avidity in metastatic disease) and ^18^F-DOPA has best performance in detecting occult metastases. ^18^FDG is good at anatomical localisation and spatial resolution, and if positive, is associated with reduced survival (Gild *et al.* 2023). Functional imaging does not correlate with Ct and CEA DT but is always negative if Ct DT is > 24 months (Kauhanen *et al.* 2011, Treglia *et al.* 2012).

## Surgery

Prophylactic, early, preventative or risk-reducing thyroidectomy in the context of MEN2 should be treated as synonyms; choice of the most appropriate term is a matter for semioticians rather than surgeons. However, defining this concept is important. Pre-operatively, prophylactic thyroidectomy is a statement of intent. Removing all C cells by means of total thyroidectomy, before MTC develops or lymph node metastases occur, is possible in patients with no biochemical, cytological and radiological evidence of MTC, a scenario most likely achievable with surgery performed within time limits predicted by individual *RET* mutations. Otherwise, if these requirements are not met, surgery should be considered therapeutic. Post-operatively, prophylactic thyroidectomy is a factual statement. Prophylactic nature of surgery is verified, in the short term, by histological diagnosis of normal thyroid, CCH or intrathyroid microcarcinoma without lymph node metastases, and in the long term, by no evidence of MTC during follow-up.

### Timing

Question of timing is relevant only to prophylactic thyroidectomy and patients with evidence of MTC should have therapeutic thyroidectomy performed immediately. Judging the optimal time for prophylactic thyroidectomy should take into account, first, genetic risk stratification of age-dependent penetrance and second, biochemical assessment of C cells reflecting their malignant transformation and increasing aggressiveness. These two considerations play out slightly differently in each of the risk groups.

All patients in *Highest risk group* (MEN2b) develop MTC and lymph node metastases early and have a worse prognosis with a 10-year survival of 75.5% compared with 97.4% in MEN2a (Modigliani *et al.* 1998). Their MTC-specific survival and outcomes are significantly inferior to patients in Moderate and High-risk groups (*P* = 0.0008, *P* = 0.0001 respectively) (Raue *et al.* 2019). Thyroidectomy within first year of life is recommended if children are diagnosed promptly (Brauckhoff *et al.* 2014, Wells *et al.* 2015, Castinetti *et al.* 2019), thyroidectomy done before and after 1 year of age led to long-term biochemical and structural remission in 83 and 15% respectively, and there was significant difference in remission status between those groups (*P* < 0.0001) (Castinetti *et al.* 2019). However, MTC-specific survival did not show significant difference between patients who had thyroidectomy before or after 1 year (Castinetti *et al.* 2019).

It has been suggested that poor prognosis in MEN2b is due to earlier onset of the disease, which can be predicted by presence of *RET* 918 mutation, rather than its aggressiveness, judged by calcitonin levels (Leboulleux *et al.* 2002). As biochemical assessment at this age is not very reliable because of physiologically high concentration of calcitonin in first 3 years of life, thyroidectomy should be considered as soon as the genetic diagnosis is made and child well enough to undergo surgery.

Timing of surgery in high and moderate risk groups can be considered together as they share certain characteristics relevant to making this decision. Although age-dependent MTC penetrance predetermined by specific *RET* mutations are different in each group, the lag time between developing MTC and progression to lymph node metastases is similar (Voss *et al.* 2017, Machens *et al.* 2021*b*) and MTC-specific survival rates and outcomes are not different in both groups (Raue *et al.* 2019). Calcitonin screening, both stimulated and unstimulated, appears to correlate well with disease progression and allows timing surgery when microcarcinomas are expected but before the likelihood of locoregional metastases (Elisei *et al.* 2013, Prete *et al.* 2021).

Active surveillance therefore has been proposed as a personalised approach aiming at avoiding operating on younger children for fear of increased risk of complications, delaying need for thyroxine replacement (noncompliance) and psychological impact of surgery on child development (Elisei *et al.* 2013, Prete *et al.* 2021). Patients in moderate group are most likely to benefit from this delaying strategy as it could be many years before thyroidectomy is deemed necessary, but it is less obvious whether delaying surgery for 2–3 years in high risk group is of great benefit. Other potential disadvantages of long-term surveillance are costs and anxiety, especially when encountering uncertain results. This can lead to additional tests and frustration resulting in patients being lost to follow-up.

It is also uncertain whether younger children have higher risk of complication than older ones. Some series reported younger children had fewer complications than older children (Machens *et al.* 2018*a*, Staubitz *et al.* 2020). Permanent hypoparathyroidism, the most prevailing complication after thyroidectomy, was linked not to patient age but performance of lymphadenectomy, the need for which might rise with age (Machens *et al.* 2018*a*, Prete *et al.* 2018). Delaying surgery also risks progression of the disease and involvement of locoregional nodes are associated with poorer prognosis and distant metastases are almost always associated with increasing nodal involvement (Machens *et al.* 2021*a*,*b*). At the end, the decision to embark on the long term active surveillance must be made with full acknowledgement that it is delaying what is inevitable for most of the patients with MEN2.

### Extent

Extent of surgery in MEN2 is concerned with the management of lymph nodes, as all carriers of pathological *RET* variant who warrant surgery should have no less than total thyroidectomy. Most importantly, every effort should be made to remove all thyroid tissue, as even small volume of remaining C cells could lead to detectable levels of calcitonin after surgery. This creates confusion about whether measurable calcitonin is caused by incomplete thyroidectomy or presence of lymph node metastases, yet not detectable by imaging. It also has an impact on defining the cure, which is variably referred to when calcitonin is either undetectable or within the normal range (Skinner *et al.* 2005, Machens *et al.* 2019). The significance of detectable but within normal range postoperative calcitonin is uncertain and has been reported in children who had only CCH or normal thyroid but not yet MTC, suggesting that incomplete thyroidectomy rather than persistent disease could sometimes be responsible for this phenomenon (Prete *et al.* 2018).

Therapeutic lymphadenectomy of appropriate central and lateral neck compartments should always be performed in patients with radiological and/or cytological evidence of lymph node metastases.

However, there is no such thing as prophylactic lymphadenectomy. There is no analogy with prophylactic thyroidectomy and the term is a misnomer. Historically, it was used when preoperative assessment showed no evidence of lymph node metastases but lymphadenectomy was performed anyway, on the assumption that the risk of metastases was significant. Therefore, preoperatively, the intent was therapeutic and postoperatively its therapeutic nature was confirmed when positive lymph nodes were found. If resected lymph nodes were not metastatic, the lymphadenectomy was not prophylactic; it was unnecessary. Results of earlier series, when ‘prophylactic lymphadenectomy’ was performed in 32–77% of patients, often with low calcitonin and normal imaging, showed that the yield of positive lymph nodes was very low and varied between 1.2 and 3.4% (Wells *et al.* 1994, Dralle *et al.* 1998, Rohmer *et al.* 2011, Prete *et al.* 2018).

The decision to perform central lymphadenectomy with curative intent in the absence of radiological and cytological evidence of lymph node involvement should be based on the appreciation of risk factors associated with potential microscopic metastases to the lymph nodes. It is a difficult decision and should be guided in the first place by *RET* pathological variant and then the size of the primary intrathyroidal tumour and levels of plasma calcitonin as well as intraoperative findings.

Patients with 918 *RET* mutation have a significant risk of lymph node metastases. MTC was found in 97% of patients operated at the median age of 14 years (range 3 months–47 years) and was limited to the thyroid in only 17% of patients for whom lymph node dissection was done (Castinetti *et al.* 2019). In another series, patients diagnosed before the mean age of 5 years had lower calcitonin levels (mean 115 ng/L) and 67% had smaller (≤10 mm) tumours but still 42% had lymph nodes and 8% distal metastases, and only 58% were biochemically cured (Brauckhoff *et al.* 2014). These findings suggest that removal of the central lymph nodes, which are expected to be the first to get involved, should be performed in most cases of MEN2b, especially in patients older than 1 year (Wells *et al.* 2015, Castinetti *et al.* 2019).

Patients in high and moderate risk groups with no radiological evidence of lymph node metastases, intrathyroidal MTC smaller than 1 cm and calcitonin levels less than 20 or 30 ng/L are unlikely to have lymph node metastases and should be considered for total thyroidectomy alone (Machens *et al.* 2009, Rohmer *et al.* 2011). Patients with intrathyroidal MTC larger than 1–1.5 cm and calcitonin levels of more than 40 ng/L are at higher risk of lymph node metastases and should be considered for central lymphadenectomy (Machens *et al.* 2009, Rohmer *et al.* 2011, Machens & Dralle 2012, Wells *et al.* 2015, Machens *et al.* 2021*c*). There is no evidence supporting lymphadenectomy in lateral neck compartments in MEN2 patients with no radiological involvement of the lymph nodes in these compartments.

Reoperations for persistent, recurrent or metastatic MTC in patients with MEN2 should be considered after thorough assessment with combined structural and functional imaging to identify tumour tissue in the neck or distal organs. The decision to proceed with further surgery must be carefully weighed against the option of observation and monitoring, especially in asymptomatic patients with elevated but not rising calcitonin and CEA levels in whom no obvious tumour is identified on imaging. Resection of locoregional or metastatic MTC should be considered in patients with well-localised disease but such operations should only be performed by surgeons with sufficient experience in such surgery and must take place in centres with the necessary multidisciplinary expertise. Treatment with tyrosine kinase inhibitors (TKIs) should be considered as a possible alternative, especially in more advanced cases when surgical cure is unlikely. RET-specific TKIs showed remarkable radiological and biochemical response rates and are an emerging effective treatment able to control disease with good tolerance and few side effects (Shankar *et al.* 2021, Hadoux *et al.* 2023).

## Complications

The risk of postoperative complications in paediatric thyroid surgery is perceived to be related to the size of the patient, complexity of surgery and experience of the surgeon (Waguespack *et al.* 2011, Wells *et al.* 2015, Prete *et al.* 2018, Machens *et al.* 2019).

Younger, smaller children are thought to be at a higher risk than older, larger ones and children in general more likely to develop postoperative complications than fully grown adults. Intuitively, perhaps this assumption makes sense, as the size of the child thyroid could be 1/10 of the size of a normal adult gland, recurrent laryngeal nerves (RLNs) smaller and parathyroid glands less visible and hidden within thymic tissue abundant in younger children. Anatomical relations of organs in a child that are still developing might differ from that of adults (Schneider *et al.* 2021, Zhang *et al.* 2022). Despite these concerns, several series reported no evidence of increased complications in younger compared to older children, indicating perhaps that the age of the child and its size is not a significant risk factor (Machens *et al.* 2018*a*, Prete *et al.* 2018, Matsushita *et al.* 2019).

There is, however, increasing evidence that the stage of the disease, which will determine the complexity and extent of surgery, is an important risk factor. Children who require total thyroidectomy alone have fewer complications in comparison to cases that need more extensive neck dissection. Central lymphadenectomy has been linked to an increased incidence of postoperative hypoparathyroidism, although in some series it did not affect the rate of this complication (Schreinemakers *et al.* 2010, Machens *et al.* 2018*a*, Prete *et al.* 2018, Matsushita *et al.* 2019).

The RLNs are at an increased risk of damage due to smaller diameter and the fact that thyroidectomy for MTC needs complete extracapsular removal for biochemical cure. The consequences of injury can be significant, leading to dysphonia, dysphagia and breathing difficulties. The reported rate of transient vocal cord paralysis in paediatric thyroidectomy ranges from 0 to 9% (Demidchik *et al.* 2006, Sosa *et al.* 2008*a*, Sinha *et al.* 2015, Hanba *et al.* 2017) but permanent injury to the RLN is rare and reported at <1.5% in most series. Managing RLNs during surgery has been aided by the use of intraoperative nerve monitoring (IONM), which has been adopted in most adult units performing high-volume thyroid surgery. The ability to perform IONM in children has only been possible with the recent advent of endotracheal electrodes (ETE) sized for children <5 years old. Other techniques for IONM include trans-cartilaginous electrode (TCE) placement, which has improved signal reliability and can be used where ETE signals fail (Zhang *et al.* 2022). In some cases, particularly in infants, TCE may be preferred over endotracheal tube placements, which are prone to intraoperative displacement. Continuous IONM is also feasible but requires mobilisation of the vagus nerve and, although this has low morbidity, it does require careful circumferential dissection of the nerve in the carotid sheath and some units would consider it unnecessary for most thyroidectomies. Proponents for continuous intraoperative nerve monitoring have shown in large series (and with regular use) it can reduce traction nerve injuries and offers an advantage in preventing such complications (Schneider *et al.* 2021). Where it certainly has a role is in the situation of extracapsular disease with involvement of the RLN, where it can aid decisions about going ahead with bilateral surgery.

Hypoparathyroidism resulting in postoperative hypocalcaemia is the commonest complication after prophylactic and therapeutic thyroidectomy for MTC in MEN2 patients. Transient hypoparathyroidism has been reported in up to 30% cases and, although parathyroid function often recovers between 3 and 6 months after surgery, permanent hypoparathyroidism (>12 months) is not uncommon and can affect as many as 14–19% of patients (Arts *et al.* 1999, Machens *et al.* 2018*a*,*b*, Prete *et al.* 2018). In children, parathyroid preservation is particularly important due to the lifetime morbidity associated with hypoparathyroidism and the use of calcium replacement. Preservation of parathyroids can be particularly challenging in those <1 year of age where the anatomical position of the parathyroid is more variable and the appearance of the gland can sometimes be difficult to distinguish from lymph nodes. Even when correctly identified, the vascular supply can be compromised given the relative size difference in vessel diameter between paediatric and adult cases. This may account for the higher rates of hypocalcaemia seen in younger patients regardless of pathology but, as mentioned above, is higher if central lymphadenectomy is performed. In a retrospective series, the difference in permanent postoperative hypocalcaemia following total thyroidectomy with and without central lymphadenectomy was 6 and 1% respectively (Moley *et al.* 2015). The auto-transplantation of parathyroid glands was, however, high (66% > 1 gland; >34% 2+ glands; 17% 3+ glands) in this series, suggesting that postoperative parathyroid function can be actively recovered with the use of auto-transplantation.

Although frequently used before the millennium, total parathyroidectomy with autotransplantation of parathyroid slivers to the non-dominant forearm or the neck has been abandoned because of its attendant 6–9% risk of permanent hypoparathyroidism. That risk compares unfavourably with the 1–4% risk attendant to *in situ* preservation of the parathyroid glands (Dralle *et al.* 1998, Moley *et al.* 2015).

The experience of the surgeon and the expertise of the team in a hospital where surgery takes place is of paramount importance and patients of all ages should be referred to centres where combined endocrine, surgical, anaesthetic and radiological experience as well as appropriate facilities contribute equally to improving postoperative outcomes (Sosa *et al.* 1998, Sosa *et al.* 2008*b*, Tuggle *et al.* 2008, Breuer *et al.* 2013).

## Phaeochromocytoma in MEN2

Phaeochromocytoma (PHAEO) is the second most frequent tumour in patients with MEN2. It is almost always located in the adrenal gland (paraganglioma <1%) and rarely malignant (1–4%) (Lee *et al.* 1996, Lora *et al.* 2005, Castinetti *et al.* 2014). The pathological process leading to the development of PHAEO is medullary hyperplasia, and consequently tumours develop multifocally within the same gland and bilaterally. It is usually diagnosed in the 3rd and 4th decade, could be symptomatic in patients with larger tumours diagnosed first or concomitantly with MTC, but in the context of known MEN2 is frequently asymptomatic and diagnosed on biochemical screening (Machens *et al.* 2013, Thosani *et al.* 2013, Lenders *et al.* 2014).

### Genetics

Most of the pathogenic *RET* variants can lead to the development of PHAEO in patients with MEN2a and MEN2b. PHAEO can also develop as part of MEN5 due to germline MAX variants, also inherited in an autosomal dominant fashion. MEN2 and MEN5 are considered under the umbrella of the PHAEO/paraganglioma cluster 2 group, which is associated with tyrosine kinase signalling. Clusters 1 and 3 genetic mutations, which include the hypoxia-signalling pathways and Wnt signalling respectively, are beyond the scope of this review (Wells *et al.* 2015, Crona *et al.* 2017).

There is a strong genotype–phenotype correlation and age-dependent penetrance (Eng & Plitt 2023, Wells *et al.* 2015, Machens *et al.* 2024). Incidence of PHEO is highest (50%) in patients with pathogenic *RET* variants 918, 883, 634 and 631, moderate (20–30%) in other *RET* mutations in codon 11, and low (10%) in remaining pathogenic variants (Wells *et al.* 2015).

Children with high-risk mutations were reported to develop PHAEO at the age of 8 and 12, and young adults with moderate risk at 19 (Nguyen *et al.* 2001, Machens *et al.* 2005, Quayle *et al.* 2007, Rowland *et al.* 2013). The mean age at presentation is between the ages of 25 and 32 (Thosani *et al.* 2013, Machens *et al.* 2013, Modigliani *et al.* 1995) and by the age of 35 and 50 years, some 50–60% of patients with high-risk and 20–36% with lower-risk mutations develop at least one PHAEO (Imai *et al*. 2013, Castinetti *et al.* 2014).

### Biochemistry and imaging

Biochemical testing and imaging of adrenals in the context of MEN2 should be considered together as they provide valuable complementary functional and anatomical information about these tumours.

Predominant adrenaline rather than noradrenaline production leads to the adrenal phenotype of PHAEO, and diagnosis is established by abnormally high levels of free plasma or fractionated urine metanephrines (Eng & Plitt 2023, Lenders *et al.* 2014, Wells *et al.* 2015). Guidelines recommend screening for PHEO to begin at 11 years for children in the ATA- high and highest-risk groups, and at 16 years in the ATA-moderate risk group (Wells *et al.* 2015). All patients scheduled for thyroidectomy must have biochemical testing before surgery to exclude PHAEO and prevent hypertensive crisis during the perioperative period.

Adrenal imaging should only be performed in patients with abnormally high catecholamines, and its aim is to assess whether PHAEO is unilateral or bilateral, and determine its multifocality, size and position within the adrenal. This information will determine whether unilateral or simultaneous bilateral surgery is appropriate and whether cortical-sparing adrenalectomy is possible. The role of imaging in the postsurgical follow-up is to identify residual or recurrent tumour and again determine the surgical approach (Timmers *et al.* 2024).

A high degree of accuracy can be achieved by combining anatomical imaging with contrast-enhanced CT or MRI, with functional imaging by positron emission tomography (PET) or single photon emission computed tomography (SPECT). Radionuclides with the best diagnostic properties in this context are ^68^Ga-DOTA-somatostatin analogues, ^18^F-FDOPA and ^18^F-FDG, with ^123^I-MIBG also able to evaluate eligibility for targeted radionuclide therapy (theranostics) (Timmers *et al.* 2024). Worldwide availability of CT scans as well as speed of whole-body scanning (a few minutes), higher spatial resolution and less motion artefacts makes it the usual first choice of investigation, often complemented by PET scanning which has better resolution, less artefacts and higher sensitivity than SPECT (Timmers *et al.* 2024).

### Surgery

Surgical resection of the adrenal gland which has developed phaeochromocytoma is the only intervention able to achieve cure. Choice of surgical strategy depends on whether PHAEO is unilateral or bilateral, presented synchronously or metachronously with other MEN2-related pathologies, and the size and anatomical location of the tumour within the adrenal. In patients diagnosed with concomitant MTC and PHAEO, adrenalectomy must always be performed first to avoid hypertensive crisis, which can be triggered by both anaesthesia and surgery (Eng & Plitt 2023, Wells *et al.* 2015).

Most patients with MEN2 are diagnosed with unilateral PHAEO and will need surgery on a single gland, but 25% have synchronous bilateral tumours and will require simultaneous surgery on both adrenals (Thosani *et al.* 2013, Castinetti *et al.* 2014). In both circumstances, minimally invasive adrenalectomy is the procedure of choice and nowadays this should be performed laparoscopically or robotically using a trans- or retroperitoneal approach (Walz *et al.* 2010, Miccoli *et al.* 2011, Bihain *et al.* 2020). Open adrenalectomy is rarely indicated but might be an option for very large tumours (>10 cm), reoperations and as a default if the minimally invasive approach fails (Ezzat Abdel-Aziz *et al.* 2015).

Historically, removal of the whole adrenal gland (total adrenalectomy, TA) was the treatment of choice. Unilateral TA had no significant metabolic consequences for the patient as the remaining adrenal would maintain normal glucocorticoid and mineralocorticoid function. However, the majority of MEN2 patients who had unilateral adrenalectomy will develop a contralateral PHEO within 10 years (Lairmore *et al.* 1993, Asari *et al.* 2006) and will need surgery on the other gland, thus being at risk of developing hypoadrenalism. The same will apply to patients requiring simultaneous bilateral surgery. Lacking steroid secretion has significant consequences, and even educated patients with optimal replacement treatments suffer from low QoL, Addisonian crises and even death (Lairmore *et al.* 1993, Asari *et al.* 2006, Hahner *et al.* 2007).

Partial adrenalectomy (PA) spares some of the adrenal tissue and aims to preserve cortical function and avoid the need for glucocorticoid and mineralocorticoid replacement (Brunt *et al.* 1996, Scholten *et al.* 2011*b*). PA is recommended by guidelines as the treatment of choice for MEN2 patients with PHAEO, although there are no prospective randomised trials comparing total and PA, and the level of evidence is low (Lenders *et al.* 2014, Wells *et al.* 2015). The volume of adrenal tissue required to maintain normal secretion and obviate the need for corticosteroid supplementation is not certain, but preservation of 10–15% of tissue preserved function in more than 80% of patients (Brauckhoff *et al.* 2008). In patients with large and multiple tumours, PA might not be possible.

Good functional outcomes can be achieved with PA, and a large international population-based study including 552 MEN2 patients showed that 57% of 114 patients who had at least one PA for PHAEO maintained normal glucocorticoid function (Castinetti *et al.* 2014). Smaller studies reported similar outcomes, with 58% of 33 MEN2 patients who had PA maintaining normal glucocorticoid function, although there is a risk of cortical function gradually declining over a period of time (Grubbs *et al.* 2013).

PA, however, cannot completely remove adrenal medulla, and development of multifocal tumours driven by hyperplasia can lead to PHAEO relapse due either to recurrence in a remnant of adrenal already operated on, which is rare (0–14%), or in the contralateral intact gland, which is more frequent (43–57%) (Lairmore *et al.* 1993, Scholten *et al.* 2011*b*, Castinetti *et al.* 2014, Korpershoek *et al.* 2014, Machens *et al.* 2022). This finding could suggest that a smaller volume of adrenal medulla reduces the risk of recurrence.

Recurrence rate after PA has been reported to be 1–11% after a mean follow-up of 6–10 years and 20% within 20 years, but we do not know if the risk of relapse is mutation specific. It is expected that longer follow-up will uncover more recurrences (Castinetti *et al.* 2016). A recent multicentre study of 256 patients from 12 countries with hereditary PHAEO showed that recurrence rate is higher after PA than TA (15 vs 4%) but mortality and metastasis rates were not different (Xu *et al.* 2024). Long-term surveillance is thus required in these patients, and recurrence after adrenal-sparing surgery should be treated by TA, or in experienced hands, by another PA (Brauckhoff *et al.* 2004).

A systematic review and meta-analysis of 25 studies compared outcomes of TA and PA for bilateral PHAEO in 1,444 patients with a variety of hereditary syndromes, of which 2/3 had MEN2. All 826 patients who had TA were steroid-dependent, while approximately 2/3 of 618 who had PA did not require steroid supplementation (*P* = 0.00001, RR 0.32). The risk of developing acute adrenal insufficiency in patients undergoing PA was almost three times lower (*P* = 0.03, OR 0.3) but the risk of recurrence was considerably higher (OR 3.72, *P* = 0.003). Risk of developing metastases (OR 1.47, *P* = 0.5), all-cause and phaeochromocytoma-specific mortality (OR1.04, *P* = 0.92 and OR 0.54, *P* = 0.53 respectively) did not differ (Zawadzka *et al.* 2023). Selpercatinib has been reported as an effective therapy against RET-mutant phaeochromocytoma (Deschler-Baier *et al.* 2025).

New intraoperative techniques, such as indocyanine green and fluorescence able to identify tumours and delineate their margins as well as blood supply to the adrenals, can potentially increase the number of PAs, preserve cortical secretion and perhaps reduce recurrence rates (Lerchenberger *et al.* 2020, Tuncel *et al.* 2021).

## Primary hyperparathyroidism in MEN2 syndrome

Parathyroids are the third endocrine organ affected in MEN2, and although multiple glands can develop adenomas or hyperplasia, it is often just one parathyroid gland overactivity which leads to the development of primary hyperparathyroidism (PHPT). PHPT in MEN2 is mostly mild and asymptomatic (Carling & Udelsman 2005, Scholten *et al.* 2011*a*), and almost always benign, with only one case of parathyroid carcinoma reported in a patient with MEN2 (Posada-Gonzalez *et al.* 2014).

### Genetics

PHPT is a very common disease globally with an overall prevalence of 0.84–0.86% (Press *et al.* 2013, Soto-Pedre *et al.* 2023), but 90% of cases are sporadic and only 10% familial. Genetic testing of patients with PHPT is increasingly used to distinguish sporadic from familial forms, and is crucial in helping to choose an appropriate surgical strategy (Bilezikian *et al.* 2022, English *et al.* 2024).

PHPT in the context of MEN2 represents just a small portion of familial cases caused by a range of syndromes such as multiple endocrine neoplasia (MEN types 1, 4, 5), hyperparathyroidism jaw-tumour (HPT-JT) and non-syndromic forms such as familial hypocalciuric hypercalcaemia (FHH1-3), neonatal severe hyperparathyroidism (NSHPT) and familial idiopathic hyperparathyroidism (FIHPT) (English *et al.* 2024). Although *RET* is included in the panel of genes for detecting familial PHPT, a large UK cohort study of 121 patients referred for genetic testing for a hereditary cause of PHPT (panel: *MEN1*, *RET*, *CDKN1A*, *CDKN1B*, *CDKN2B*, *CDKN2C*, *GCM2*, *CASR*, *GNA11* and *AP2S1*) reported that 16% of patients had a pathogenic variant, the most common being *CASR (58%)* and *MEN1 (32%)*, with no RET mutations identified (Mariathasan *et al.* 2020). This finding can be explained by the MEN2 prevalence being ten times lower (13–24/1,000,000) in comparison to MEN1 (1–3/100,000) and FHH (74/100,000) (Dershem *et al.* 2020, Al-Salameh *et al.* 2021, Mathiesen *et al.* 2022), and the fact that penetrance of PHPT in MEN2, depending on *RET* mutation, is only 5–30% and almost 100% in MEN1 and FHH. A large multicentre study of 1,085 patients with MEN2A found that only ten (0.9%) cases presented initially with PHPT, and nine of these ten patients were found to have synchronous MTC (Larsen *et al.* 2020). These two studies imply that the pick-up rate for diagnosing pathogenic RET mutations causing MEN2A syndrome in patients presenting only with PHPT is low, and that screening for RET mutations in this scenario may not be helpful (English *et al.* 2024).

Development of PHPT in the context of MEN2 shows, like for other target organs but perhaps less strongly, genotype–phenotype correlation, and about 95% cases are caused by activating mutations in codons 10 and 11. Mutations in cysteine residues C630 and C634 in codon 11 are associated with the majority of PHPT cases, and 87% of them are at codon 634 with the highest penetrance at C634R (Mulligan *et al.* 1994, Eng *et al.* 1996, Raue & Frank-Raue 2012, Mathiesen *et al.* 2017). Mutations in cysteine residues C609, C611, C618 and C620 in codon 10 are responsible for 2–12% of cases (Herfarth *et al.* 1996, Frank-Raue *et al.* 2011, Raue & Frank-Raue 2012). Mutations at codon 14 (V804) and 15 (C891) are also implicated, but PHPT is never associated with the pathological *RET* 918 variant causing MEN2b.

Accordingly, ATA Guidelines classify these pathogenic mutations as high-risk of developing PHPT, with penetrance of 30% for codon 634 and low-risk 10% for all others (Wells *et al.* 2015). Penetrance of PHPT in MEN2 is age-related, with 9.4–19.1% of patients with high-risk mutations developing hyperparathyroidism between the ages of 30–40 years, with penetrance of 14% by age 30 years, 26% by age 40 years, 48% by age 60 years and 81% by age 70 years (Schuffenecker *et al.* 1998, Machens *et al.* 2013). Penetrance of PHPT in patients with low-risk mutations ranges from 1.3 to 2.7% between the ages of 46–54.5 years, with penetrance of 0% by age 20 years, 0.5% by age 30 years, 1.8% by age 40 years, 3.9% by age 50 years and 3.9% by age 60 years (Asari *et al.* 2006, Frank-Raue *et al.* 2011, Frank-Raue *et al.* 2013, Machens *et al.* 2013).

### Biochemistry and imaging

Although a large amount of information about genotype–phenotype correlation in patients with MEN2 is available, there is a dearth of data on biochemical and imaging assessment which specifically relates to patients with this syndrome who developed PHPT. Considering the rarity of MEN2, low penetrance of PHPT in this condition, and the fact that only some of the patients will require surgery, this gap in evidence is unlikely to disappear any time soon, if ever.

It is therefore reasonable and generally accepted that biochemical assessment of patients with MEN2 aiming at establishing the function of the parathyroid glands should follow recommendations for sporadic PHPT and include measuring albumin-corrected or ionised plasma calcium, intact parathormone, vitamin D levels, renal function and 24 h urine collection for calcium. Biochemistry is used for screening, establishing diagnosis of PHPT and deciding whether surgical intervention is necessary, as well as defining outcomes of surgery. According to ATA Guidelines, screening for PHPT should only be performed in patients with mutations associated with this condition and should start at the age of 11 and 16 years for high- and low-risk mutations respectively. Earliest reported cases of PHPT were 2, 6, 7 and 10 years old; there perhaps might be a case for screening to start at the age of 5 (Wells *et al.* 2015). Diagnostic threshold for PHPT, defined by above normal levels of plasma calcium and PTH together with evidence of hypercalciuria, renal and skeletal damage, as well as patients’ symptoms, should be considered together when deciding whether parathyroid surgery is indicated, similarly to patients with sporadic PHPT (Bilezikian *et al.* 2022).

Imaging of parathyroid glands in patients with MEN2 is not essential in cases with proven PHPT who are scheduled to undergo total thyroidectomy. Four glands visualisation during operation, aided with intraoperative IOPTH monitoring, can correctly identify abnormal glands, and their removal should result in cure (Shawky *et al.* 2019, Shawky *et al.* 2020, Kurzawinski *et al.* 2024). Imaging of parathyroids with US, CT or nuclear scanning (MIBI*, *choline*, *methionine) is, however, essential if PHPT is diagnosed after thyroid surgery has been performed some time ago. Parathyroid imaging in patients with sporadic and familial PHPT is helpful, and meta-analyses of different methods showed sensitivity of 63–71% for US, 81% for 3D CT scans, 66–88% for MIBI and 69% for C-methionine, but is lower in recurrent disease (Minisola *et al.* 2016, Treglia *et al.* 2018).

### Surgery

Surgical considerations regarding management of parathyroid glands in patients with MEN2 fall into three distinctive clinical scenarios. First, very unlikely for the reason we have discussed above, is when a patient diagnosed initially with PHPT is also subsequently found to have MEN2. Second, is enacted when the MEN2 patient with the RET mutation known to be associated with PHPT is scheduled to undergo prophylactic or therapeutic thyroidectomy and either has normal biochemistry indicating physiological parathyroid function or his calcium and PTH levels are diagnostic of PHPT. The third scenario is encountered when PHPT is diagnosed some years after thyroidectomy has already been performed, not an unusual situation given that RET genotypes associated with PHPT have much earlier penetrance of MTC and later penetrance of PHPT.

Historically, the operation of choice, frequently also applied in other forms of familial PHPT, was a total parathyroidectomy with either autotransplantation of fragmented parathyroid tissue into muscle in the neck or forearm of the non-dominant upper limb, or subtotal parathyroidectomy with at least one or half a gland *in situ* preserved on a vascular pedicle. This approach has gradually fallen out of favour when surgeons realised that this strategy does not necessarily prevent recurrence of PHPT, quoted at 9–18% for total and 9–33% for subtotal parathyroidectomy, but is associated with a very high rate of postoperative permanent hypoparathyroidism, the overall risk of which is 9–33% and 14–66% respectively for each approach (O'Riordain *et al.* 1993, Raue *et al.* 1995, Herfarth *et al.* 1996, Kraimps *et al.* 1996, Dotzenrath *et al.* 2001, Twigt *et al.* 2013, Holm *et al.* 2023). Published series suggest that alternative approaches of identifying and resecting only enlarged and macroscopically abnormal parathyroid glands have a similar rate of PHPT recurrence of 14–33% but lower rate of hypoparathyroidism of 14–19% (Holm *et al.* 2023, Twigt *et al.* 2013, Dotzenrath *et al.* 2001, Kraimps *et al.* 1996, Herfarth *et al.* 1996, Raue *et al.* 1995, O'Riordain *et al.* 1993).

Bilateral neck exploration, as well as minimally invasive parathyroidectomy, can be used in patients with recurrent PHPT depending on the ability to localise abnormal parathyroids with imaging. Intraoperative PTH monitoring technique, which relies on a short biological half-life of PTH (5 min) and predicts biochemical cure when excision of pathological parathyroid leads to a 50% reduction of PTH concentration, is very likely to be helpful in this scenario and carries a promise of better outcomes and increased use of MIP (Shawky *et al.* 2019, Shawky *et al.* 2020, Graceffa *et al.* 2022). The role of other perioperative adjuncts such as autofluorescence and ICG angiography fluorescence of parathyroid glands is promising but still needs to prove their value in parathyroid surgery (Hauck *et al.* 2022).

## Conclusions

Caring for patients with MEN2 is a multidisciplinary effort but timely and adequate surgical resection of tumours associated with this syndrome is the only treatment able to achieve long lasting cure. Recent scientific discoveries and technological advances have made the surgical decision process more precise and facilitated personalised treatments. *RET* analysis enables us to see this syndrome not as a monolith but as a cluster of different phenotypic presentations, each sending the patient on an individual journey, which can be anticipated but not determined. Biochemical monitoring provides regular updates on transformation of endocrine cells in target endocrine organs and, together with imaging, helps to decide on timing and extent of surgery. Advances in surgical technology allow for safer and less invasive interventions resulting in fewer complications, less trauma and better functional outcomes.

But for the operation to be successful, it must be performed by an experienced surgeon with not only thorough knowledge of anatomy and skill in surgical techniques, but also in possession of a deep understanding of MEN2 as a whole, including the genetics and molecular biology of this condition. Such knowledge helps when making instant and correct intraoperative decisions about the number of parathyroids to be removed *(usually only one)*, performing lymphadenectomy *(sometimes)* or leaving behind adrenal *(desirable)* or thyroid tissue *(never!!!)*. Importantly, it will also allow the surgeon to make the difficult but informed decision of not embarking on futile operations *(rare but crucial)*.

Calibrating the magnitude of surgery able to cure but do minimal harm, timing and performing it well is the art of surgical precision in MEN2 patients. Surgical outcomes have improved in the last 30 years and we need to continue on this road. Precision in surgery aiming at near perfect surgical performance is achievable but it must be performed in centres with expertise and facilities which can maximise opportunities offered by precision medicine. Identifying such centres globally will not only improve outcomes but also accelerate future research, both to the benefit of patients with MEN2.

## Footnote

This review has been written in the context of the *Endocrine-Related Cancer* themed collection RET@Thirty, which celebrates the 30th anniversary of identifying *RET* as a causative gene of MEN2. We have focused specifically on the surgical issues of MEN2 with the full knowledge that other multifaceted aspects of this ‘chameleon gene’ such as *RET* biology, structure, signalling, genetic testing and oncology are discussed expertly in 12 other reviews, and for a full update on *RET*, ideally, the whole ERC themed collection should be read together.

## Declaration of interest

The authors declare that there is no conflict of interest that could be perceived as prejudicing the impartiality of the work reported.

## Funding

This work did not receive any specific grant from any funding agency in the public, commercial or not-for-profit sector.
